# Novel agents and regimens for acute myeloid leukemia: 2009 ASH annual meeting highlights

**DOI:** 10.1186/1756-8722-3-17

**Published:** 2010-04-23

**Authors:** Xiongpeng Zhu, Yuehua Ma, Delong Liu

**Affiliations:** 1 Department of Hematology, First Hospital of Quanzhou Affiliated to Fujian Medical University, Quanzhou, 362000, China; 2 Division of Hematology and Oncology, New York Medical College, Valhalla, NY 10595, USA

## Abstract

Prognostic markers, such as NPM1, Flt3-ITD, and cytogenetic abnormalities have made it possible to formulate aggressive treatment plans for unfavorable acute myeloid leukemia (AML). However, the long-term survival of AML with unfavorable factors remains unsatisfactory. The latest data indicate that the standard dose of daunorubicin (DNR) at 45 mg/m^2 ^is inferior to high dose 90 mg/m^2 ^for induction therapy. The rates of complete remission and overall survival are significantly better in the high dose induction regimen. New regimens exploring the new liposomal encapsulation of Ara-C and DNR as well as addition of gemtuzumab ozogamicin monoclonal antibody have been studied. New agents, including the nucleoside analogues (clofarabine, sapacitabine, elacytarabine), FLT3 inhibitor (sorafenib), farnesyl-transferase inhibitor (tipifarnib), histone deacetylase inhibitor (vorinostat), lenalidomide, as well as DNA methyltransferase inhibitors (decitabine, azacitidine), were recently reported for AML treatment in the 2009 ASH annual meeting. This review also summarizes the updates of the clinical trials on novel agents including voreloxin, AS1413, behenoylara-C, ARRY520, ribavirin, AZD1152, AZD6244, and terameprocol (EM-1421) from the 2009 ASH annual meeting.

## Introduction

Acute myeloid leukemia (AML) is the most common type of acute leukemia in adults. Over the past twenty years, the studies on the pathogenesis and prognosis of AML have made revolutionary progress. However, only one-third of adult AML can be cured even to this date. The treatment of refractory, relapsed and elderly AML remains a major challenge. In recent years, new regimens and novel agents are being studied in an effort to improve complete remission (CR) rate and overall survival. This study will review the latest advances in AML treatment and summarize the highlights from the 2009 ASH Annual Meeting.

## New regimens for induction therapy of newly diagnosed AML

### High dose daunorubicin improves survival

The standard induction regimen for newly diagnosed AML consists of daunorubicin (DNR) 45 mg/m^2 ^intravenously for 3 days and cytarabine (AraC) 100 mg/m^2 ^by continuous infusion for 7 days [1]. With this regimen 60% to 80% of young adults and 40% to 60% of older adults can achieve a CR.

Several major studies, particularly Cancer and Leukemia Group B (CALGB) 9621 [2,3] and the French ALFA 9000 studies [4], have shown that higher doses of DNR (80 or 90 mg/m^2^) can be administered safely. Recently, there are two major prospective studies compared DNR 90 mg/m^2 ^with 45 mg/m^2 ^in the induction regimen. Eastern Cooperative Oncology Group (ECOG) studied 657 AML patients between the age of 17 to 60 [5]. The study showed significantly higher CR rate for patients receiving 90 mg/m^2 ^(70% versus 57%). More importantly, overall survival (OS) was significantly prolonged (23.7 vs 15.7 months). The Dutch-Belgium Hemato-Oncology Cooperative Group (HOVON)/Swiss Group for Clinical Cancer Research (SAKK) compared DNR 90 mg/m^2 ^versus 45 mg/m^2 ^in 813 patients older than 60 years [6]. The results showed that CR rate was 64% and 54% respectively, while CR rate after only one course of treatment was 52% and 35% respectively. The OS rate was not significantly different for the whole group. However, for the patients between the age of 60 to 65, the OS rate was significantly better in the high dose group (38% vs 23%). The rates of serious adverse events were similar in the two treatment groups in both studies.

Based on historic trials and the most recent prospective studies, Rowe points out that 45 mg/m^2 ^of DNR should no longer be the standard-dose for induction therapy [7]. Instead, for induction therapy of all age groups, DNR dose should be between 60 mg/m^2 ^to 90 mg/m^2 ^for 3 days, but the exact optimal dosage remains to be established.

### New formulations of old agents

Liposomal encapsulation of drugs can reduce the toxicity and decrease drug doses with controlled-release effect. CPX-351 is a liposomal formulation that encapsulates cytarabine and daunorubicin at a 5:1 molar ratio. A recently completed phase 1 study recommended that 90-minute infusions of 101 u/m^2 ^be given on days 1, 3, and 5 (1 u = 1 mg Ara-C + 0.44 mg DNR) [8]. The results showed that liposomal encapsulation of this chemotherapy doublet changed the safety profile by reducing non-hematologic toxicities including hair loss, gastrointestinal toxicities and hepatic toxicity, while retaining hematopoietic cytotoxicity. A phase IIb randomized study was initiated to compare CPX-351 with conventional DA regimen (Ara-C + DNR) in AML patients aged 60-75. CPX-351 exhibits an acceptable safety profile for use in older, newly diagnosed AML patients[9].

### Targeted therapy regimens

In recent years, encouraging results have been achieved by using monoclonal antibodies for targeted therapy of the solid and hematologic malignancies. CD33 antigen is expressed in more than 90% of AML cells, while expression in normal tissue is very weak. Gemtuzumab ozogamycin (GO) is chemoimmunotherapy agent consisting of a monoclonal antibody against CD33 conjugated to calichemycin. GO triggers apoptosis when hydrolyzed in the leukemic blasts. GO has been approved by the U.S. FDA for the treatment of the elderly (> 60 years) with AML in first relapse [10]. Standard induction regimen with or with out GO were compared in a randomized study which enrolled 1115 younger adults with AML. The results showed a similar CR rate in both arms, but a significantly improved DFS among patients receiving GO--51% versus 40% at 3 years (*P *= .008)[11].

GO + chemotherapy is also used in AML with special chromosome abnormalities. GO + FLAG has been used to treat 34 cases of newly diagnosed AML younger than 60 with core binding factor (CBF) abnormality [Inv(16) = 10; t(8;21) = 24]. The induction regimen consisted of the following agents: Fludarabine 30 mg/m^2^/d, d1-5, Ara-C 2 g/m^2^/d, d1-5, GO 3 mg/m^2^/d1, and G-CSF 3 mg/kg/d. The GO-FLAG regimen in CBF^+ ^AML yielded impressive clinical and molecular response in 29 of the 34 patients[12].

A phase II study of My-FLAI aiming to assess toxicity and efficacy was done in patients with newly diagnosed AML aged more than 60 years. Fifty-one patients were enrolled with a median age of 68 years. Twenty-five patients had a secondary AML and 31% had a complex karyotype. Fludarabine (25 mg/m^2^), cytarabine (1 g/m^2^), and idarubicin (5 mg/m^2^) were administered for three consecutive days. GO (5 mg) was infused at day four. Twenty-seven patients achieved a CR and 4 obtained a partial response for an overall response rate (ORR) of 61%. The results showed that the four drug regimen My-FLAI was well tolerated in an elderly AML population, but its efficacy did not appear to be superior to that of standard "3+7" regimen[13].

## New regimens for refractory/relapsed AML

High-dose cytarabine (HiDAC) is commonly used for induction of relapsed or refractory AML. At the 2009 ASH meeting, Sarah et al reported a novel, timed-sequential regimen that takes advantage of synergy when mitoxantrone is given after cytarabine [14]. It was a retrospective analysis of patients with relapsed or refractory high-risk AML. Those patients received HiDAC/mitoxantrone regimen, with cytarabine at 3 gm/m^2 ^over four hours on days 1 and 5 plus mitoxantrone at 30 mg/m^2 ^over one hour immediately following the HiDAC on days 1 and 5. HiDAC/mitoxantrone induction was well tolerated and demonstrated an overall response rate of 55% with induction death rate of 9%.

To further enhance the CR rate in refractory/relapsed AML, the Japanese Adult Leukemia Study Group (JALSG) reported a phase II study of FLAGM (Fludarabine + High-Dose Ara-C + G-CSF + mitoxantrone) in 41 patients with relapsed or refractory AML. The patients were treated with fludarabine 15 mg/m^2 ^twice daily (d1-4), Ara-C 2 g/m^2 ^(d1-4), G-CSF 300 μg/m^2 ^(d1-4), and mitoxantrone 10 mg/m^2 ^(d3-5). FLAGM yielded a 70% response rate in either relapsed or refractory AML patients. Although randomized studies are still needed, FLAGM appears to be a good option for the treatment of either relapsed or refractory AML patients [15].

Thomas et al conducted a retrospective analysis of response (CR and CRi) and survival for patients with first relapsed AML treated with either IHDAraC or IHDAraC + GO regimen [16]. Univariate analysis showed that IHDAraC +GO induction, as compared with IHDAraC, was associated with a better response rate (68% vs 48%, p = 0.08), a lower relapse rate (31% vs 66%, p = 0.02), a better overall survival (median 35 months vs 19 months, p = 0.02) and a better event free survival (median not reached vs 10 months, p = 0.02).

## New Agents

### Nucleoside analogues

Nucleoside analogues transform into active metabolites (triphosphate nucleoside analogues) in the cells and inhibit DNA synthesis. Clofarabine is a new nucleoside analogue, a potent inhibitor of both ribonucleotide reductase and DNA polymerase. At the 2009 ASH meeting, a few studies on clofarabine were reported, either clofarabine alone or in combination with low-dose Ara-C, or high-dose Ara-C with the monoclonal antibody GO in the treatment of elderly AML or relapsed AML [17-21]. Two novel nucleoside analogues, sapacitabine and elacytarabine, were also reported for the therapy of the elderly with refractory or relapsed AML [22,23] (Table 1).

**Table 1:** Nucleoside analogues in clinical trials

Study Agents	Other agents	Disease	Dosage	Clinical trails	No Pts	Response	**Reference**
Clofarabine	HD Cytarabine,	Relapsed and refractory AML	22,5 mg/m^2 ^i.v qd, d1-5 GO 6 mg/m^2 ^d6	Phase II	20	CR 50%	[17]
Clofarabine		Elderly AML	20-30 mg/m^2^ i.v qd, d1-5	Phase II	112	CR 33-56%	[18]
Clofarabine	HD Cytarabine	Relapsed and refractory AML	25 mg/m^2^/d 1-5d	Phase I II	38	CR 45%	[19]
Clofarabine	GO	Relapsed and refractory AML	20 mg/m^2^/d or 30 mg/m^2^/d d1-5	Phase I	14	MTD: 20 mg/m^2 ^clofarabine	[20]
Clofarabine	LD Cytarabine	Elderly untreated AML	20 mg/m^2 ^i.v qd, d1-5	Phase II	40	CR 59% CRp 6%	[21]
Sapacitabine		Elderly Relapsed and refractory AML	200 or 300 mg po bid ×7d, 400 mg po bid ×3d/w ×2w	Phase II	60	CR 10%	[22]
Elacytarabine		Relapsed and refractory AML	2,000 mg/m2 CIV d1-5q3w	Phase II	61	CR 15%	[23]

Abbreviations: GO: gemtuzumab ozogamycin; HD: high dose; LD, low dose; CRp: CR without platelet recovery; MTD: maximal tolerated dose;

In a preliminary study, twenty patients with relapsed/refractory AML were enrolled to receive a regimen including intermediate dose Ara-C, clofarabine and GO [17]. The preliminary results was 10 of 20 (50%) patients achieved a complete remission, 1/20 a partial response, 7/20 had resistant disease, 2/20 died of complications during the aplastic phase. Further studies are warranted (Table 1).

In a single-arm, multi-center, phase II, open-label trial, 112 patients of previously untreated AML, ≥ 60 years old, and with at least one unfavorable prognostic factor were enrolled to receive single agent clofarabine [18]. In patients ≥ 70 y (n = 69), ORR was 39%, CR 33%; In patients with unfavorable cytogenetics(n = 62), ORR was 42%, CR 32%. Patients with 2 unfavorable prognostic factors (n = 45) had ORR of 51%. Patients with 3 unfavorable factors (n = 40) had ORR 38%. Patients ≥ 70 with intermediate or unfavorable karyotype (n = 25) had ORR 48% and CR 40%; in patients ≥ 70 with unfavorable karyotype (n = 9) ORR and CR were 56%. Patients ≥ 70 with both AHD and unfavorable karyotype (n = 18), ORR was 33% and CR 22%. In patients ≥ 70 with AHD and intermediate karyotype (n = 8), ORR and CR were 63% (Table 1). It therefore appears that single agent clofarabine has reasonable activity in newly diagnosed elderly AML patients.

There was another report of a phase II trial which enrolled 38 patients with relapsed or refractory AML. The patients received a regimen with G-CSF priming, clofarabine and high dose Ara-C (GCLAC) [19]. The CR was 45% and the CR +CRp rate was 64%. These rates were 50% CR and 65% CR+CRp among 1^st ^salvage patients (95% CI 41-85%), respectively, and 70% CR + CRp excluding patients who relapsed after allogeneic SCT (Table 1). It is important to point out that the relatively higher CR rate could be in part due to the higher dose of AraC.

Clofarabine was tested in a phase I, dose escalation study in fourteen patients with relapsed and refractory AML, who received clofarabine in combination with fractionated GO in 2 cohorts. The MTD of clofarabine in combination with fractionated GO is 20 mg/m^2^/day for 5 days [20] (Table 1).

Forty patients with AML were enrolled in a phase II study to receive clofarabine plus low-dose Ara-C induction followed by consolidation with clofarabine plus low-dose Ara-C alternating with decitabine. Of the 34 patients evaluable for response, 20 (59%) achieved CR and 2 (6%) CRp for an overall response rate (ORR) of 65%. The therapy achieves high response rate with a manageable toxicity profile and low induction mortality in elderly patients with previously untreated AML [21](Table 1).

### FLT3 inhibitors (Fms-like tyrosine kinase 3 inhibitors)

The Flt3-internal tandem duplication (ITD) can be found in approximately 30% of all AML patients and confers a poor risk status characterized by an increased relapse rate and poor overall survival [24]. Moreover, Flt3-ITD-positive AML patients relapsing after allogeneic stem cell transplantation (SCT) have very limited therapeutic options. Sorafenib is a multikinase inhibitor that is approved for the treatment of metastatic renal cell and hepatocellular carcinoma. A questionnaire was developed and sent to 28 centers in Germany in order to obtain more insight into the clinical efficacy and tolerability of sorafenib monotherapy in Flt3-ITD positive AML. Of the 18 patients treated with sorafenib, five were primary refractory to induction chemotherapy and 13 were in first (n = 11) or second (n = 2) relapse. Patients received between 200 mg and 800 mg sorafenib p.o. daily. The median treatment duration was 98 days (range, 16-425 days). All patients achieved a hematological response (HR) characterized by complete (n = 16) or near complete peripheral blast clearance (n = 2). After a median treatment duration of 180 days (range, 82-270 days), 7 of 18 (39%) patients developed clinical resistance. Therefore, sorafenib monotherapy has significant clinical activity in Flt3-ITD positive relapsed and refractory AML [25].

In addition, combination therapy with sorafenib was shown to be effective in reducing mutant clones in patients with FLT3 mutations but was not able to completely eradicate them. These data suggest that sorafenib can achieve temporary disease control, but should be integrated into induction and consolidation regimens to achieve maximal outcome [26-28] (Table 2).

**Table 2:** FLT3 inhibitors in clinical trials

Study Agents	Other agents	Disease	Dosage	Clinical trails	No Pts	Response	Reference
Sorafenib		Flt3-ITD+ Relapsed and refractory AML	200 mg-800 mg qd	retrospective	26	CHR: 88%	[25]
Sorafenib	as part of induction therapy and salvage	FLT3^+^AML, untreated Relapsed		retrospective	128	CR/CRp: 7%	[26]
Sorafenib		FLT3^+^AML Relapsed and refractory	800 mg q.d	retrospective	8	CR: 25%	[27]
Sorafenib	Idarubicin, cytarabine	FLT3^+^AML untreated	400 mg po bid ×7 d	Phase II	18	CR/CRp: 94%	[28]

Abbreviations: CR: complete remission; CRp: CR without platelet recovery; CHR: complete hematological response

Another retrospective study analyzed sorafenib treatment in 128 patients [26]. Among these patients, twenty-three patients (18 FLT3-WT, 5 FLT3 mutated) received FLT3 inhibitors as part of their induction and 9 of them achieved either CR (n = 6) or CRp (n = 3). These results suggest that therapy with FLT3 inhibitors has the potential to improve the outcome of patients with FLT3 mutations (Table 2). Prospective study is needed to confirm the findings.

In another clinical study, sorafenib was evaluated in 8 AML patients with FLT3+ either prior to or after allogeneic stem cell transplantation (allo-SCT) [27]. Two of four patients who received sorafenib for refractory/relapsed AML after allo-SCT achieved complete remission (CR), the other two pts had hematological response. The rest four patients were treated prior to allo-SCT. Two of the four relapsed patients showed response to sorafenib treatment, thereby permitting allo-SCT. One of these two patients achieved HR, the other had regression of multiple isolated cutaneous manifestations. Sorafenib treatment was well tolerated (Table 2).

In a phase II study, eighteen patients with newly diagnosed AML and mutated FLT3 were enrolled to receive sorafenib, idarubicin, and Ara-C [28]. 94% of the patients achieved a morphological CR/CRp and 6% achieved PR. This regimen was found to be effective in reducing the mutant clones (Table 2).

In summary, sorafenib appears to provide a useful option for treatment of relapsed/refractory AML patients. However, large prospective study is needed to confirm the results from the small observational studies.

### Farnesyl-transferase inhibitor (FTI)

In recent years, studies have shown that Ras gene mutation plays an important role in leukemogenesis [29]. By inhibiting farnesyl protein transferase, FTI prohibits the Ras protein farnesylation, schizolysis and carboxyl methylation, thus disrupting the critical Ras signaling pathway.

A phase II study assessed the efficacy and toxicity of tipifarnib-bortezomib combination in 80 AML patients >18 years, unfit for conventional therapy, or >60 years, in relapse (Table 3). Nine patients (11%) achieved CR, 1 patient had PR, and in 2 cases an hematological improvement (HI) was documented for an overall response rate (ORR) of 19%. Tipifarnib (± bortezomib) may represent an important option in a subset of high risk/frail AML patients [30].

**Table 3:** Farnesyl-transferase inhibitor in clinical trials

inhibitors	Other agents	Disease	Dosage	Clinical trails	No Pts	Response	Reference
Tipifarnib	Bortezomib 1.0 mg/m^2^	Elderly or relapsed AML	300-600 mg bid, ×21d	Phase II	80	CR: 11%	[30]
Tipifarnib	+/- etoposide	Elderly untreated AML		Phase II	107	Safe	[31]

Feldman et al compared efficacy of tipifarnib +/- oral etoposide with traditional cytarabine/anthracycline-based induction regimen in older patients with AML. The results suggest that better CR did not translate into better survival outcomes (median OS 6.2 vs 7.7 months; p = 0.82 by log-rank test) [31].

### Histone deacetylase inhibitors

Vorinostat is a new anti-cancer agent inhibiting histone deacetylase and has been shown to have some efficacy in treatment of AML [32-34]. Vorinostat in combination with idarubicin and ara-C has synergistic antileukemia activity in a sequence dependent fashion [35,36]. A phase II study of vorinostat in combination with idarubicin and cytarabine as front line therapy for AML or MDS patients was reported (Table 4). This study enrolled 52 pts at the time of the report, and 45, all with AML, are evaluable for response (median age 53 yeas (range 19-65). The CR after one course of therapy was achieved in 35 pts and 1 pt achieved a CRp with incomplete platelet recovery for an overall response rate of 80%. Seven (15%) pts did not respond to therapy. Therefore, the combination of vorinostat, idarubicin and cytarabine is safe and active in AML[37]. CR or CRi was achieved by 18% pts with MDS, 8% with relapsed/refractory AML, and 36% with untreated AML; and HI was reported in 9% pts with MDS, 4% with relapsed/refractory AML, and 8% with untreated AML.

**Table 4:** Histone deacetylase inhibitors in clinical trials

Study Agents	Other agents	Disease	Dosage	Clinical trails	No Pts	Response	Reference
Vorinostat	Idarubicin, Cytarabine	untreated AML	500 mg po tid d1-3	Phase II	52	CR/CRp: 80%	[37]
Vorinostat	decitabine	untreated, relapsed AML	400 mg qd, po 1-7d or 1-14d	Phase I/II	72	MTD not reached	[38]

Abbreviations: CR: complete remission; CRp: CR without platelet recovery; MTD: maximal tolerated dose;

There was also a preliminary report of a Phase I, open-label, multicenter, dose-escalating study, designed to determine the maximum-tolerated dose (MTD) vorinostat combined either concurrently or sequentially with decitabine in patients (pts) with AML/MDS. 72 patients were enrolled. CR or CRi (CR with incomplete count recovery) was achieved by 18% pts with MDS, 8% with relapsed/refractory AML, and 36% with untreated AML. Thus, the combination of vorinostat with decitabine, either concurrently or sequentially, is possible without significant toxicity, and shows activity in MDS and untreated AML[38].

### DNA Methyltransferase inhibitors

Decitabine inhibits DNA methyltransferase, leading to DNA hypomethylation and cell differentiation or apoptosis. A combination of decitabine and GO was found to be effective with low side effects in previously untreated or refractory/relapsed AML patients, especially in elderly patients[39]. In this phase II study, 33 previously untreated patients with AML/high-Risk MDS were enrolled to received GO with decitabine. 24% of the patients had CR/CRp. Five (15%) patients had clearance of marrow blasts and 1 patient had hematological improvement (hemoglobin). The toxicities were minimal and the regimen can be safely delivered to older patients (Table 5). In a retrospective study, 79 patients with relapsed or refractory AML received decitabine/GO combination. 34% patients responded: 16% CR; 5% CRp; 13% PR-[40]. It is noteworthy that the response rates from these two studies are similar to that of the single agent GO, and therefore could be mainly due to the activity of GO (Table 5)

**Table 5:** DNA Methyltransferase inhibitors in clinical trials

Study Agents	Other agents	Disease	Dosage	Clinical trails	No Pts	Response	Reference
decitabine	GO	Elderly, untreated AML	20 mg/m2 IV ×5d, GO 3 mg/m^2 ^IV × 1 d 5	Phase II	33	CR/CRp: 42%	[39]
decitabine	GO	Relapsed and refractory AML	20 mg/m2 IV ×5d	retrospective	79	CR/CRp: 21%	[40]
Azacytidine		Elderly relapsed and refractory AML	75 mg/m^2^/d IV, d1-7	retrospective	184	CR/CRi: 10%	[41]
Azacytidine	gemtuzumab ozogamicin (GO)	high-risk AML	75 mg/m^2^/d IV, d1-7	retrospective	56	CR/CRi: 10%	[42]
Azacytidine	Bortezomib	Relapsed and refractory AML	75 mg/m^2^/d IV, d1-7	Phase I	23	CR/CRi: 21%	[43]

Abbreviations: GO: gemtuzumab ozogamycin; CR: complete remission; CRi: CR with incomplete count recovery; CRp: CR without platelet recovery; MTD: maximal tolerated dose;

The French ATU program performed a retrospective analysis of 184 patients with refractory or relapsed AML who received azacytidine [41]. 11% of the patients responded (7%CR,3%CRi,1%PR). It appears that single agent azacytidine has only limited activity in AML patients relapsed or refractory to intensive frontline therapy (Table 5).

Combination of azacitidine with bortezomib or low-dose GO was also studied in relapsed or refractory AML patients [41-43] (Table 5).

In a retrospective analysis, 56 patients with poor-risk AML/MDS received treatment with azacitadine and low-dose GO. 27% of the patients achieved a CR/CRi. An additional seven patients cleared their peripheral blood blasts or had hematologic improvement but did not have remission [42] (Table 5).

In a phase I study, 23 patients with relapsed or refractory AML were enrolled to receive bortezomib and 5-azacytidine. The response rate was 26% (6/23) (3-CR, 2-CRi, and 1-PR). The combination of 5-azacytidine and bortezomib was well tolerated and appeared to be active in this cohort of relapsed or refractory AML patients [43] (Table 5).

In a phase I dose-finding trial, twenty eight patients with AML/MDS were enrolled to receive vorinostat plus azacitidine (AZA) in 8 cohorts [44]. Surprisingly, 53% of the patients achieved CR. In particular, 10 of 12 high-risk MDS/AML patients (83%) went into CR. This combination was found to be well tolerated in repetitive cycles. The optimal dose of AZA in this regimen appears to be 55 mg/m^2^. Phase II study is being done.

### Novel agents in early clinical development

#### Voreloxin

Voreloxin is a first-in-class anticancer quinolone derivative that intercalates DNA, inhibits topoisomerase II, and induces apoptosis. A preliminary report on a voreloxin trial revealed clinical activity in previously untreated elderly (age ≥ 60) AML patients who are unlikely to benefit from standard chemotherapy[45]. In this phase II dose optimization study, 105 patients were treated, with 93 patients evaluable. The CR + CRp rate of the 3 dose schedules was 41%, 29%, 38%, respectively. ORR across the 3 schedules was 35%; (Table 6). The study is still ongoing.

**Table 6:** Novel agents in clinical trials

Study Agents	Other agents	Disease	Dosage	Clinical trails	No Pts	Response	Reference
CPX-351 (liposomal cytarabine: daunorubicin at 5:1)		Elderly untreated AML	100 u/m^2 ^D1,3,5	Phase IIb	45	acceptable toxicity and comparable to standard regimen	[9]
Voreloxin (anticancer quinolone derivative)		Elderly untreated AML	72 mg/m^2 ^qw × 3	Phase II	105	CR/CRp: 47%	[45]
Amonafide (AS1413)	Cytarabine	Secondary AML	600 mg/m^2^/d IV d1-5	Phase II	88	CR/CRi: 42%	[46]
behenoylara-C	Idarubicin Etoposide	untreated AML	300-350 mg/m^2^ × 10d	Phase II	165	CR: 87%	[47]
Lenalidomide	Cytarabine, Daunorubicin	5q-AML/MDS	10 mg/d po d1-21	Phase I/II	18	CR/CRi: 47%	[50]
Ribavirin	Cytarabine	AML			13	CR:7%	[51]
ARRY-520	idarubicin	refractory AML	0.8-5.6 mg/m2 IV	Phase I	33	MTD: 4.5 mg/m2	[52]
AZD1152		relapsed AML	50 mg-1600 mg	Phase I/II	64	MTD: 1200 mg	[53]
AZD6244		AML	100 mg po bid	Phase II	47	Feasible treatment	[56]
Terameprocol		relapsed and refractory AML	1000-2200 mg iv, 3/w, ×2-3w	Phase I/II	16	MTD: 1500 mg	[57]

Abbreviations: CR: complete remission; CRi: CR with incomplete count recovery; CRp: CR without platelet recovery; MTD: maximal tolerated dose;

#### Amonafide L-malate (AS1413)

Amonafide L-malate (amonafide, AS1413) is a unique DNA intercalator. In a phase II study, 88 patients with secondary AML were enrolled to receive amonafide and Ara-C. Overall CR + CRi rate was 42%. CR rates among age <60 and ≥ 60, was 39.4% and 43.6%, respectively; among tAML and prior MDS, 40% and 44.2%, respectively; for patients with intermediate and unfavorable cytogenetics, the CR rates were 61.1% and 23.8%, respectively (Table 6). This study showed that amonafide in combination with cytarabine produced a high complete remission rate and durable responses in both older and younger patients with secondary AML[46].

#### Behenoylara-C

Behenoylara-C has three-phosphoryl in the fourth N of Ara-C, making it more lipophilic than Ara-C. Its concentration is maintained longer in the blood (especially blood cells) and tissues. This agent is transformed into Ara-C in the liver, spleen, kidney and leukemia cells, which inhibits DNA synthesis. Taiichi et al studied 165 patients with untreated AML using the combination of behenoylara-C and idarubicin. 86.7% of the patients had CR. The patients with good or intermediate risk factors had remarkable improvements. The study showed that the treatment is effective and safe [47] (Table 6).

#### Lenalidomide

Lenalidomide is one of the three new drugs approved by the U.S. FDA to treat MDS [48,49]. Treatment of 5q-low-risk MDS with LEN can achieve high rate of cytogenetic CR. In a recent phase II study of LEN in combination with Ara-C and daunorubicin in high risk MDS/AML with del 5q, 28% responded (Table 6). The results show that LEN combined with chemotherapy in AML treatment is feasible, without significant additional toxicity[50].

#### Ribavirin

The eukaryotic translation factor, eIF4E, is overexpressed in AML, and is associated with poor prognosis. Ribavirin is clinically used as an antiviral molecule, and its structure is similar to the m(7)G cap of mRNA, thus inhibiting eIF4E-induced export and translation of sensitive transcripts. Assouline et al carried out the first clinical trial targeting eIF4E with ribavirin in combination with AraC in AML patients (Table 6). Clinical and molecular efficacy has been evaluated in 13 patients. The treatment was well tolerated by all patients. No hemolytic anemia was seen. There was one complete remission, two partial remissions, two blast responses and four patients with stable disease. Unfortunately, all patients eventually acquired resistance to therapy and eventually relapsed. Hence, the novel therapies combined with ribavirin are being sought to overcome resistance and prolong remission[51].

#### ARRY-520

The kinesin spindle protein (KSP) plays a major role for the assembly of a normal bipolar spindle and is also required for cell cycle progression through mitosis. ARRY-520 is a potent, selective inhibitor of KSP. Thirty-three patients with AML were enrolled to receive different schedule of ARRY-520: 15 in the single-dose schedule (dose levels 2.5, 3.75, 4.5 and 5.6 mg/m^2^) and 18 in the divided dose schedule (dose levels 0.8, 1.2, 1.5 and 1.8 mg/m^2^/day). The maximal tolerated dose (MTD) was 4.5 mg/m^2 ^for the single-dose schedule with the dose-limiting toxicity (DLT) of grade 3 mucositis. The MTD was 1.5 mg/m^2^/day (cumulative dose per cycle of 4.5 mg/m^2^) for the divided dose schedule, with DLTs being grade 3 mucositis, hand-foot syndrome and hyperbilirubinemia. ARRY-520 was well tolerated. Four of 33 patients (12%) showed at least 50% reduction in bone marrow blasts (Table 6). Therefore, ARRY-520 showed promising clinical activity and was well tolerated in both schedules[52].

#### AZD1152

Aurora B kinase plays a major role in regulating mitosis and is overexpressed in AML. AZD1152 is a highly potent and selective inhibitor of aurora B kinase. It has been shown to inhibit tumor growth in vivo. A phase I/II study was conducted to assess the safety and efficacy of AZD1152 in patients aged >18 years with advanced AML (Table 6). The MTD of AZD1152 was defined as 1200 mg in patients with relapsed AML, and an overall clinical response rate (CR+CRi+PR) of 23% was observed [53].

#### AZD6244

AZD6244 is one of the orally bioavailable small molecule inhibitors of MEK kinase [54,55]. AZD6244 was studied in 47 relapsed or refractory AML in a phase II multicenter clinical study [56]. Among these patients, *FLT3 *ITD or TKD mutation was positive in 10, negative in 36, mutational status was unknown in 1. Median number of prior therapies for AML and/or MDS was 2 (range, 0-6). The AZD6244 dose was 100 mg twice daily; 42 pts were evaluable. Median number of cycles administered was 1 (range, 1-9). AZD6244 related serious adverse events included fatigue, nausea and dehydration, occurring in 7%, 5% and 5%, respectively. Minor responses were seen, no CR was reported. The study showed that the oral MEK inhibitor AZD6244 is tolerable in AML. Further investigation of AZD6244 in combination with drugs that target other critical signaling/transcriptional pathways in AML is being considered.

#### Terameprocol

The inhibitor of apoptosis protein (IAP), survivin, is a key regulator of cell cycles. In leukemic cells, survivin is involved in leukemia cell survival and resistance to chemotherapeutics and Flt-3 inhibitors. A clinical trial with terameprocol (EM-1421), a novel survivin and cdc2 (CDK1) inhibitor, was done in patients with advanced hematological malignancies (Table 6). In a phase I dose-finding trial, 16 patients with advanced, relapsed or refractory hematological malignancies were treated with 1000, 1500 or 2200 mg of intravenous terameprocol 3×/week (wk) for 2 of 3 wks. The MTD was found to be 1500 mg 3×/week for 2 of 3 wks [57].

## Conclusions and future directions

Prognostic markers, such as NPM1, Flt3-ITD, and cytogenetic abnormalities have made it possible to prospectively formulate aggressive treatment plans for unfavorable AML. However, the long-term survival of AML with unfavorable factors remains unsatisfactory. Combination of azacytidine and vorinostat showed surprisingly high response rate. Prolonged survival without curing high risk MDS/AML patients with azacytidine therapy suggests that disease modification instead of cure of AML patients may be an alternative goal of treating elderly patients not suitable for aggressive therapy. New regimens and novel agents targeting specific pathways reviewed in this report may bring AML treatment into a new era.

## Competing interests

The authors declare that they have no competing interests.

## Authors' contributions

XZ and DL are involved in concept design. All authors participated in data collection, drafting and critically revising the manuscript.
